# Flexible support material maintains disc height and supports the formation of hydrated tissue engineered intervertebral discs in vivo

**DOI:** 10.1002/jsp2.1363

**Published:** 2024-08-05

**Authors:** Alikhan B. Fidai, Byumsu Kim, Marianne Lintz, Sertac Kirnaz, Pravesh Gadjradj, Blake I. Boadi, Maho Koga, Ibrahim Hussain, Roger Härtl, Lawrence J. Bonassar

**Affiliations:** ^1^ Meinig School of Biomedical Engineering Cornell University Ithaca New York USA; ^2^ Sibley School of Mechanical and Aerospace Engineering Cornell University Ithaca New York USA; ^3^ Department of Neurological Surgery, Weill Cornell Medical College New York‐Presbyterian Hospital New York New York USA

**Keywords:** 3D printing, regenerative medicine, spine, total disc replacement

## Abstract

**Background:**

Mechanical augmentation upon implantation is essential for the long‐term success of tissue‐engineered intervertebral discs (TE‐IVDs). Previous studies utilized stiffer materials to fabricate TE‐IVD support structures. However, these materials undergo various failure modes in the mechanically challenging IVD microenvironment. FlexiFil (FPLA) is an elastomeric 3D printing filament that is amenable to the fabrication of support structures. However, no present study has evaluated the efficacy of a flexible support material to preserve disc height and support the formation of hydrated tissues in a large animal model.

**Methods:**

We leveraged results from our previously developed FE model of the minipig spine to design and test TE‐IVD support cages comprised of FPLA and PLA. Specifically, we performed indentation to assess implant mechanical response and scanning electron microscopy to visualize microscale damage. We then implanted FPLA and PLA support cages for 6 weeks in the minipig cervical spine and monitored disc height via weekly x‐rays. TE‐IVDs cultured in FPLA were also implanted for 6 weeks with weekly x‐rays and terminal T2 MRIs to quantify tissue hydration at study endpoint.

**Results:**

Results demonstrated that FPLA cages withstood nearly twice the deformation of PLA without detrimental changes in mechanical performance and minimal damage. In vivo, FPLA cages and stably implanted TE‐IVDs restored native disc height and supported the formation of hydrated tissues in the minipig spine. Displaced TE‐IVDs yielded disc heights that were superior to PLA or discectomy‐treated levels.

**Conclusions:**

FPLA holds great promise as a flexible and bioresorbable material for enhancing the long‐term success of TE‐IVD implants.

## Graphical abstract

Here, we report the assessment of degradable materials to support tissue engineered intervertebral discs. We demonstrate that flexible PLA is superior in maintaining disc height and supporting tissue formation in vivo.

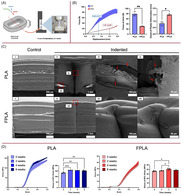

## 1 INTRODUCTION

Developing a clinically functional tissue‐engineered intervertebral disc (TE‐IVD) with sufficient mechanical function has been a challenge for two decades. Robust mechanical function immediately following implantation is crucial for the long‐term viability of TE‐IVDs. As such, several advancements have been made to enhance the stabilization of implanted tissues while recapitulating the complex architecture of native disc. The first composite TE‐IVD was implanted subcutaneously in athymic mice and was comprised of a polyglycolic acid scaffold seeded with annulus fibrosus (AF) cells and an alginate core seeded with nucleus pulposus (NP) cells.^1^, ^2^ Years later, another group engineered a composite disc‐like angle‐ply implant with an agarose core encapsulated by electrospun layers of polycaprolactone.^3^ These studies demonstrated that composite TE‐IVDs bear a striking histological, morphological, mechanical, and biochemical resemblance to native disc. However, subcutaneous implantation does not accurately recapitulate the biologically challenging IVD microenvironment.

To more accurately mimic the avascular and nutrient‐deficient IVD microenvironment, later studies implanted composite TE‐IVDs in the caudal and lumbar spines of athymic rats.^4^, ^5^, ^6^ These implants remained in the spine for up to 6 months, demonstrating the long‐term feasibility of implanting engineered tissues. Furthermore, histological analysis revealed good integration between TE‐IVDs and adjacent vertebrae. The functional integration between TE‐IVDs and neighboring vertebrae is essential for facilitating nutrient transport into the disc and transmitting mechanical loads.^7^ Although these studies addressed the biologically challenging IVD microenvironment, mechanical loading in the rat spine is substantially different than loading in humans.^8^, ^9^ In humans, the IVD endures cyclic loading in compression, shear, and torsion.^10^, ^11^ Therefore, an appropriate animal model for testing TE‐IVDs must subject these implants to similar loading.

More recently, studies in porcine, canine, caprine, and bovine models have gained interest for testing the functionality of TE‐IVDs.^12^, ^13^, ^14^, ^15^ Large animals possess IVDs that are more anatomically, structurally, and mechanically similar to human discs.^16^, ^17^, ^18^ Despite notable advantages of these models, new challenges arise in the fixation of implants in the spine. Migration of the implant or complete collapse of the disc space upon loading remains an enduring roadblock to integration and clinical translation.^12^, ^13^ To address these challenges, several studies have employed TE‐IVD support structures to preserve disc height and provide initial mechanical support. Titanium fixation plates were previously used to prevent implant migration in the goat cervical spine.^14^ Although these plates enhanced implant retention, they necessitate an additional surgery to remove them once integration is achieved. Another study demonstrated that a resorbable plating system comprised of poly(lactic‐co‐glycolic acid) (PLGA) preserved disc height and enhanced implant retention in the canine cervical spine.^19^ A resorbable system circumvents the need for a secondary surgery by degrading at a timescale that would facilitate implant integration. However, a key commonality between both systems is that they prioritized choosing a material with high stiffness to preserve disc height. Although stiffer materials are superior for transmitting spinal loads, they can lead to various failure modes including fracture, integration failure, and loosening, which collectively lead to implant failure.^20^, ^21^, ^22^ Consequently, there remains a critical need to assess the importance of stiffness versus elasticity when selecting an optimal material for mechanically augmenting TE‐IVDs.

A novel material that has gained interest as an attractive candidate for mechanically augmenting TE‐IVDs is FlexiFil (FPLA). FPLA is an elastomeric and bioresorbable thermoplastic co‐polyester 3D printing filament that is amenable to the fabrication of TE‐IVD support structures. By leveraging results from a recently published finite element (FE) model of the minipig cervical spine, we can engineer anatomically accurate FPLA support cages for total disc replacement.^12^ Furthermore, recent work demonstrated that FPLA scaffolds were non‐cytotoxic and did not impede the deposition of glycosaminoglycans from NP cells.^23^ This study also demonstrated that FPLA possesses superior stability against hydrolysis when compared to PLA, preventing brittle fracture following 26 weeks of degradation under physiologically relevant conditions.^23^

Although FPLA holds great promise as a flexible TE‐IVD support material, no present study has evaluated the capacity of a flexible material to prevent implant fracture, enhance implant retention, and support the maturation of engineered tissues in a large animal model. With this in mind, the goals of this study were to (1) analyze failure modes of flexible and stiff TE‐IVD support cages under mechanical loading, (2) assess the ability of flexible support materials to preserve disc height and prevent implant migration, (3) demonstrate the ability of a flexible material to support the formation of hydrated TE‐IVDs in the minipig spine.

## 2 MATERIALS AND METHODS

### 2.1 3D printing of TE‐IVD support cages

To accurately capture the dimensions of the minipig's cervical disc, micro‐computed tomography scans of the C3‐4 and C5‐6 levels in the Göttingen minipig spine were obtained as previously described.^12^ One‐piece cage models were drafted in Inventor Professional 2020. Cages were 3D printed on a Prusa i3 MK3S+ 3D printer (Prusa3D, Prague, Czech Republic) out of poly(lactic acid) (PLA) (*n* = 15) and FPLA (*n* = 15) (FormFutura, Nijmegen, Netherlands). Cages were printed using an E3D 0.25 mm nozzle with 100% infill.

### 2.2 Force–displacement indentation of cage materials

We have previously demonstrated via FE modeling that minipig vertebrae possess anatomic features that generate stress concentrations on TE‐IVD support cages in vivo.^12^ To assess the mechanical response of cages to these stress concentrations and to simulate the sharpest geometry found on adjacent vertebrae, FPLA (*n* = 3) and PLA (*n* = 3) cages were subjected to force–displacement testing with a wedge indenter (United Testing Systems, Model FM‐20, New Castle, DE). Support cages were mounted to a block of polypropylene and loaded along the midplane at 0.1 mm/s to achieve quasi‐static conditions until reaching a depth of 2 mm with a sampling rate of 50 Hz. An indentation depth of 2 mm was chosen to simulate a failure mode predicted by our previously published FE model of the minipig spine. At 2 mm of displacement, our model predicted collapse of the cage due to rigid body contact.^12^

### 2.3 Scanning electron microscopy (SEM) of indented cages

To visualize the failure modes of support cages from indentation, SEM images of control and indented FPLA (*n* = 3) and PLA (*n* = 3) cages were analyzed for resulting microscale damage. Cages were fixed to 18 mm aluminum specimen mounts with double‐sided tape and sputter‐coated with gold/palladium alloy for 25 s at a target current of 30 mA.^24^, ^25^ Samples were imaged on a TESCAN Mira3 SEM (TESCAN, Kohoutovice, Czech Republic).

### 2.4 Uniaxial compression of cages after immersion

To assess the effects of physiologically relevant conditions on implant mechanics, PLA (*n* = 12) and FPLA (*n* = 12) cages were incubated in PBS at 37°C for 0 (*n* = 3), 2 (*n* = 3), 4 (*n* = 3), and 6 weeks (*n* = 3). Cages were uniaxially compressed under quasi‐static conditions between flat plates using a Screw‐drive, uniaxial tension/compression tester (United Testing Systems, Model FM‐20), with a triangular waveform at 0.1 mm/s and sampling frequency of 50 Hz.^12^ Cages were compressed until reaching 2 mm of displacement, or 80% strain.

### 2.5 IVD cell isolation and culture

Whole IVDs were dissected from Meishan pigs as previously described.^26^ Tissue was washed in PBS (Dulbecco's 1× PBS, Corning, NY) with 1% Antibiotic Antimycotic Solution (Corning 100× AbAm, Corning, NY) and separated into AF and NP sections. AF and NP tissue was cut into smaller tissue fragments and digested overnight at 37°C in 150 mL and 125 mL of 0.3% wt./vol. collagenase type II (Worthington Biochemical Corporation, Lakewood, NJ), respectively. Primary cells were collected from digested tissue using 100 μm nylon filters (Corning, NY) and washed with PBS + 1% AbAm. The resulting cell populations were seeded at 8000 cells/cm^2^ and expanded in 2D culture in growth media comprised of Ham's F‐12 (Corning, NY), 10% fetal bovine serum (FBS, Gemeni Bio, West Sacramento, CA), 1% AbAm, and 25 μg/mL ascorbic acid (Sigma‐Aldrich, St. Louis, MO). Cells were cultured to 70%–80% confluence at 37°C with 5% CO_2_ under normoxic conditions and with media replenished three times a week.

### 2.6 TE‐IVD fabrication

Cells were removed from culture flasks using 0.25% trypsin with 2.21 mM EDTA once sufficiently confluent (Corning, NY). NP cells were encapsulated in 3% (wt./vol.) low viscosity grade alginate (NovaMatrix, Wilmington, DE) mixed in a 2:1 ratio with 0.02 g/mL CaSO_4_. The resulting 10 × 10^6^ cells/mL alginate solution was set between two glass plates to achieve a sheet gel with a height of 3 mm. The resulting sheet gel was then soaked in a 60 mM solution of CaCl_2_ to crosslink for 1 h. Once crosslinked, NP plugs were generated from the sheet gel using a 4 mm biopsy punch. A seeding density of 10 × 10^6^ cells/mL was selected for the NP to promote greater matrix synthesis and account for the reduced biosynthetic activity of NP cells.^27^ FPLA cages were placed in 6‐well plates and NP plugs were placed in the center of individual cages. A collagen‐AF solution was created by mixing type I collagen sourced from rat‐tail tendons (BioIVT, Westbury, NY) with a basic working solution (10× PBS, 1× PBS, 1 M NaOH).^4^ The neutralized collagen solution was seeded with AF cells at a density of 10 × 10^6^ cells/mL to achieve a final concentration of 10 mg/mL. The resulting cellular collagen gel was pipetted around NP plugs and within cages to form the AF. Implants were then cultured at 37°C for 1 h to allow for gelation. Once gelled, 5 mL of growth media were added to each well. Media was replenished three times a week over a culture period of 18 days.

### 2.7 In vivo study design

**FIGURE 1:**
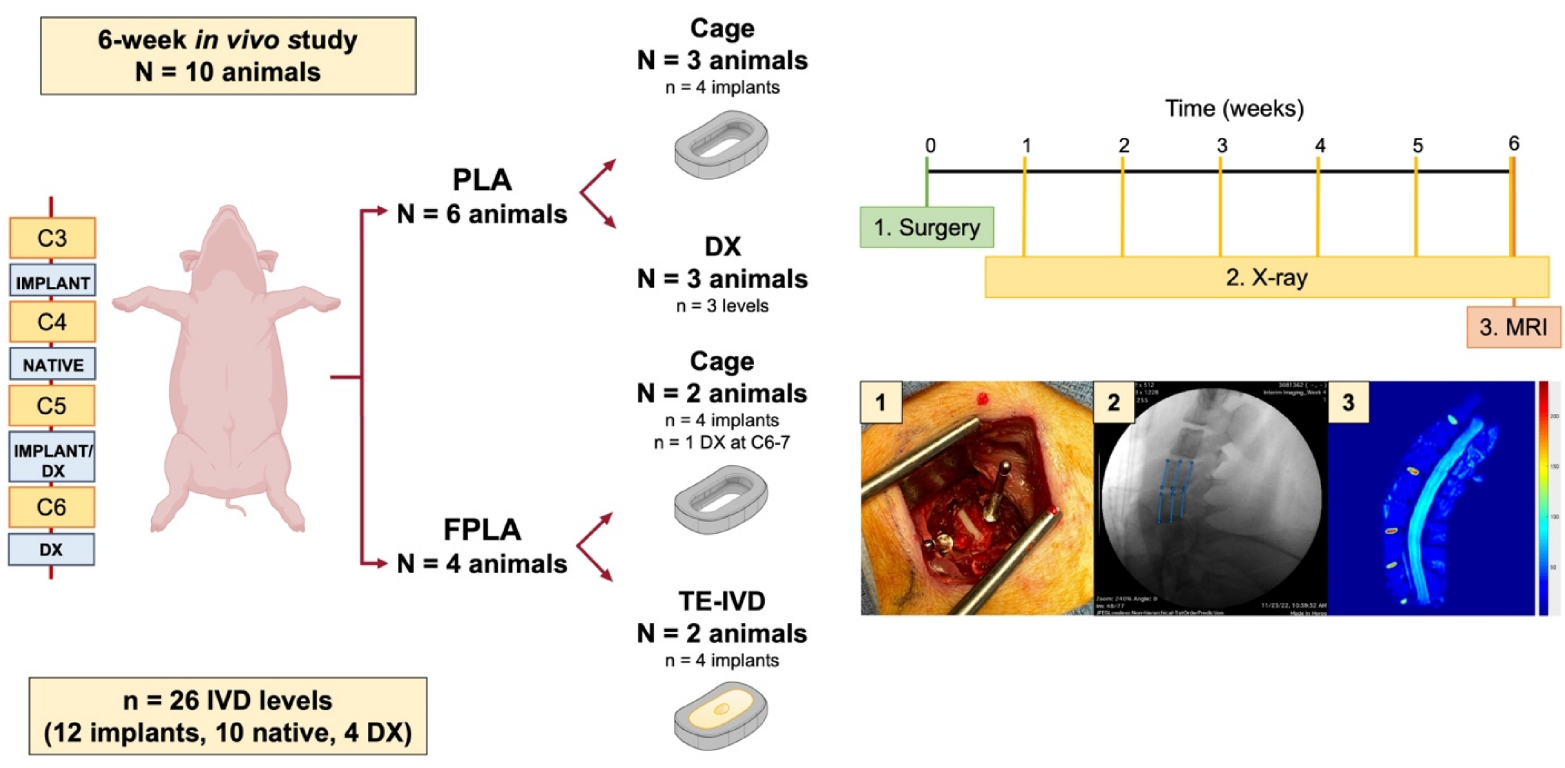
In vivo study design and outcome measures. (Left) A total of 10 minipigs received a complete discectomy (DX) followed by implantation of PLA cages (*n* = 4), FPLA cages (*n* = 4), or TE‐IVDs cultured in FPLA (*n* = 4) for up to 6 weeks. (Right) All animals received a complete DX at study baseline. Weekly x‐rays were taken until study endpoint to monitor disc height maintenance. T2 MRIs were taken at study endpoint to quantify hydration of implanted TE‐IVDs.

To assess whether stiff or flexible materials are better suited for maintaining disc height in vivo, we implanted support cages comprised of PLA (*n* = 4) or FPLA (*n* = 4) in skeletally mature Göttingen minipigs for 6 weeks.^13^ The Göttingen minipig was selected as our animal model due to anatomic similarities in the curvature of the cervical spine. Both human and minipig cervical spines are lordotic in curvature. Moreover, minipig spines possess IVDs with comparable stiffness values in compression and shear.^16^, ^17^, ^28^, ^29^ TE‐IVDs cultured in FPLA support cages (*n* = 4) were also implanted to determine if FPLA could support the formation of hydrated tissues in vivo. At study baseline, minipigs received a complete discectomy (DX) to resect any native disc tissue at C3‐4, C5‐6, and C6‐7. Animals received TE‐IVD support cages or TE‐IVDs cultured in FPLA cages at C3‐4 and C5‐6. C4‐5 was left untouched as a native control and C6‐7 served as a DX control. X‐rays were taken each week to monitor implant stability and disc height over time. T2 MRIs were taken at study endpoint to quantify tissue hydration of implanted TE‐IVDs relative to native, cage, and DX levels (Figure 1).

### 2.8 Total discectomy in porcine cervical segments^13^

Skeletally mature Göttingen minipigs (*n* = 10) were obtained from Marshall BioResources. All animals were 2–3 years old at the time of surgery. All experimental procedures were reviewed and approved by the Institutional Animal Care and Use Committee at Weill Cornell Medicine. Animals were housed at Skirball Center for Innovation (Orangeburg, NY) during the duration of the study. For DX, all animals underwent general anesthesia which was induced with an IV ketamine/midazolam cocktail and maintained with a combination of IV fentanyl/lidocaine/ketamine and inhaled isoflurane. Following endotracheal intubation, the animals were placed in dorsal recumbency with the neck hyperextended and secured to the table with adhesive tape. The surgical site was prepared by clipping hair and scrubbing with chlorhexidine and betadine scrub solution. Cefazolin was given intravenously (antibiotic prophylaxis) 30 min before the incision which was repeated every 2–3 h thereafter during the surgical procedure. A ventral midline incision from the base of the larynx to the sternum was carried out to reach the cervical spine. Subplatysmal dissection was performed and the medial border of the paired sternocephalicus and sternohyoideus muscles were separated with Metzenbaum scissors and blunt dissection. The carotid sheath was identified by palpation and retracted laterally, and the trachea and esophagus were retracted medially. Self‐retaining retractors were placed to maintain exposure. The prevertebral fascia was identified and intraoperative fluoroscopy was used to localize and confirm the operative levels with a spinal needle at C3‐4 and C5‐6. The prevertebral fascia was then dissected with a hemostat and peanut sponge to expose the disc space The longus colli was elevated bilaterally using monopolar cautery for additional exposure. A Caspar distraction system with self‐drilling pins and retractors was placed in the upper and lower vertebral bodies of the operative levels to open the disc space. The disc was incised with an #11‐blade scalpel along the endplates and laterally to the uncovertebral joints. A complete DX was then performed using a combination of curettes, pituitary rongeur, and Kerrison rongeurs down to the posterior longitudinal ligament (PLL). The PLL was entered with a nerve hook and then resected with Kerrison rongeurs, exposing the underlying dura.

### 2.9 In vivo total disc replacement using TE‐IVD

After 18 days, TE‐IVDs were removed from culture and transported sterilely to the operating room. Empty PLA and FPLA cages were transported to the operating room under the same conditions. TE‐IVDs cultured in FPLA (*n* = 4), PLA cages (*n* = 4), or FPLA cages (*n* = 4) were implanted at C3‐4 or C5‐6 in the minipig spine after complete DX. Four levels were left untreated following surgery to serve as a discectomy control and four levels at C4‐5 did not undergo surgery to serve as a native control. For treatment groups, a trial cage was placed in the disc space initially to determine the ideal size of implant for the disc space. After the ideal size of the implant was determined, FPLA cages (with or without TE‐IVD) or PLA cages were slipped down and inserted into the space. Distraction was slowly released, and implant stability was ensured under direct visualization. The distraction pins were removed. Hemostasis was achieved using bipolar cauterization and hemostatic matrix. A multilayer wound closure was performed with absorbable sutures. Immediate postoperative x‐rays were taken. After surgery, animals were recovered from anesthesia. Eating habits, ambulatory activities, health status, and neurological functions were monitored postoperatively.

### 2.10 Disc height index (DHI) measurements

X‐rays were taken each week following implantation and until euthanasia. Animals were euthanized after 6 weeks or upon cage fracture. Terminal disc height measurements were taken from x‐ray images at sacrifice. DHIs were calculated using a method previously described.^30^ DHI measurements of DX or implant‐treated segments were normalized to adjacent healthy discs.

### 2.11 Quantitative magnetic resonance imaging

To determine disc hydration, T2 relaxation times at control and implant levels were quantified, as previously described.^30^ This method segments the NP from surrounding tissues by applying a Gaussian mixture model to first‐echo T2 images spanning two adjacent vertebrae. This separates intensities into two distinct populations: NP tissue or surrounding bone and soft tissue. The NP is then segmented by thresholding voxels three standard deviations above the average surrounding tissue's relaxation time to generate an NP mask. The mask is then applied to a quantitative T2‐mapped image to generate a T2 heatmap, where hotter colors indicative of greater T2 intensity. The mean relaxation time of DX or implant‐treated segments was normalized to adjacent healthy discs.

### 2.12 Data analysis and statistics

Differences in indented cage mechanics and yield behavior were assessed via unpaired *t*‐tests with Welch's correction. Differences in immersed cage mechanics were assessed using one‐way ANOVAs with Tukey's multiple comparisons tests. Analysis of cage and TE‐IVD DHIs was performed using a one‐way ANOVA. All statistical analysis confirmed by Tukey's HSD (α = 0.05).

## 3 RESULTS

### 3.1 FPLA resisted microscale damage from indentation

**FIGURE 2:**
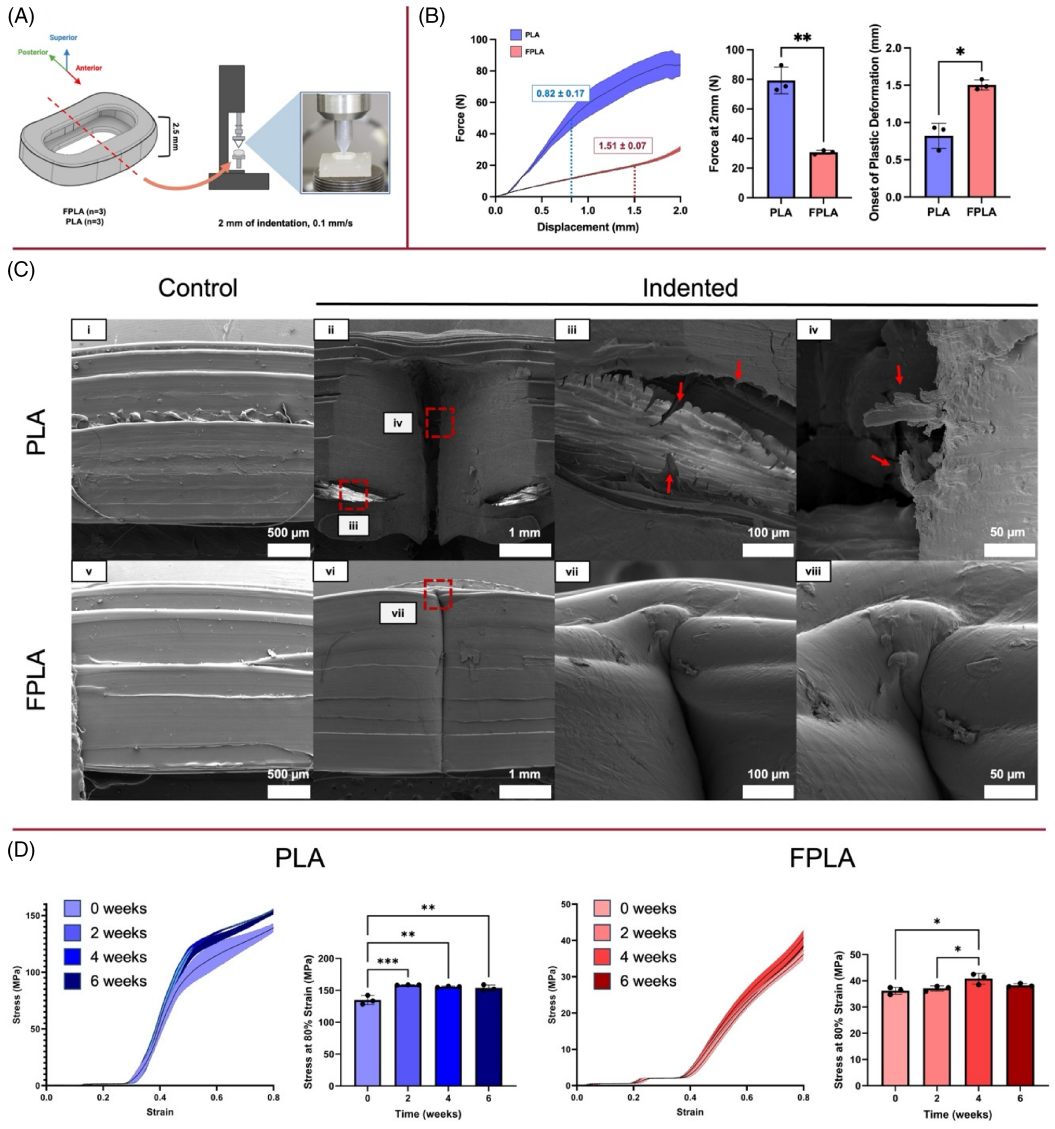
Assessment of cage mechanics and resulting microscale damage. (A) Indentation schematic of PLA (*n* = 3) and FPLA (*n* = 3) cages. (B) FPLA cages experienced significantly less force at 2 mm of indentation than PLA cages (*p =* 0.01). FPLA cages plastically deformed at significantly higher displacements than PLA cages (*p =* 0.0109). (C) Indentation led to delamination of print layers and brittle fracture in PLA cages (red arrows). FPLA cages resisted microscale damage from indentation. (D) Immersion of cages led to significant increases in the stress at 80% strain. PLA cages experienced significantly greater stress after 2 (*p =* 0.0006), 4 (*p =* 0.0014), and 6 (*p =* 0.0026) weeks of immersion. FPLA cages experienced significantly greater stress after 4 weeks of immersion when compared to the 0‐week (*p =* 0.0139) and 2‐week (*p* = 0.0420) time points.

Mechanical testing revealed that FPLA is more compliant and has a greater elastic region than PLA. To simulate the same stress concentrations validated by our FE model of the porcine cervical spine, a wedge indenter was loaded at the midplane of empty cages (Figure 2A **)**. At 2 mm of displacement, PLA cages experienced an average force of 79.3 ± 9 N whereas FPLA cages experienced an average force of 30.8 ± 1.4 N, yielding a 2.5‐fold decrease in force (*p* = 0.001). This result indicates that at the same displacement, FPLA cages experience significantly less stress than PLA. To determine the onset of plastic deformation, the maximum change in slope was calculated following linear elastic behavior. PLA cages began to plastically deform after 0.82 ± 0.17 mm of displacement. Meanwhile, FPLA cages plastically deformed after 1.51 ± 0.07 mm of displacement, leading to a nearly two‐fold increase in extensibility (*p =* 0.0109) (Figure 2B).

FPLA resisted microscale damage from indentation to prevent delamination of print layers and brittle fracture seen in indented PLA. To visualize damage, SEM images of control and indented cages were taken (Figure 2C). Images of control PLA (i) and FPLA (v) cages were indistinguishable, with no notable deformations and uniform print layers. Upon indentation, PLA cages were left with a wide band of plastic deformation stemming from the indentation site (ii). Meanwhile, FPLA cages were left with a narrow band of plastic deformation (vi). Further investigation of indented PLA revealed delamination of print layers, yielding long drawn‐out fibers extending from the bulk material (iii). Meanwhile, indentation did not lead to delamination in FPLA cages (vii). Closer inspection of indented PLA revealed evidence of brittle fracture, resulting in jagged edges and roughening of the adjacent bulk material (iv). In contrast, indented FPLA folded in on itself, preserving the integrity of the bulk material (viii).

Uniaxial compression of cages immersed in PBS at 37°C revealed that immersion led to small, but significant increases in the stress at 80% strain for both cage materials (Figure 2D). To simulate in vivo conditions, PLA (*n* = 12) and FPLA (*n* = 12) cages were incubated in PBS at 37°C for 0 (*n* = 3), 2 (*n* = 3), 4 (*n* = 3), and 6 weeks (*n* = 3). At 0 weeks, PLA cages experienced an average stress of 134.69 ± 7.08 MPa at 80% strain. Meanwhile, FPLA cages experienced an average stress of 36.18 ± 1.34 MPa at 80% strain. Immersion of PLA cages for 2 (*p =* 0.0006), 4 (*p =* 0.0014), and 6 weeks (*p =* 0.0026) led to significant increases in the stress at 80% strain when compared to the 0‐week control. Immersion of FPLA cages for 4 weeks led to significant increases in the stress at 80% strain when compared to the 0‐week (*p* = 0.0139) and 2‐week (*p* = 0.0420) timepoints. Immersion of PLA cages led to greater changes in cage mechanics than immersed FPLA.

### 3.2 FPLA cages and stably implanted TE‐IVDs restored native disc height in vivo

**FIGURE 3:**
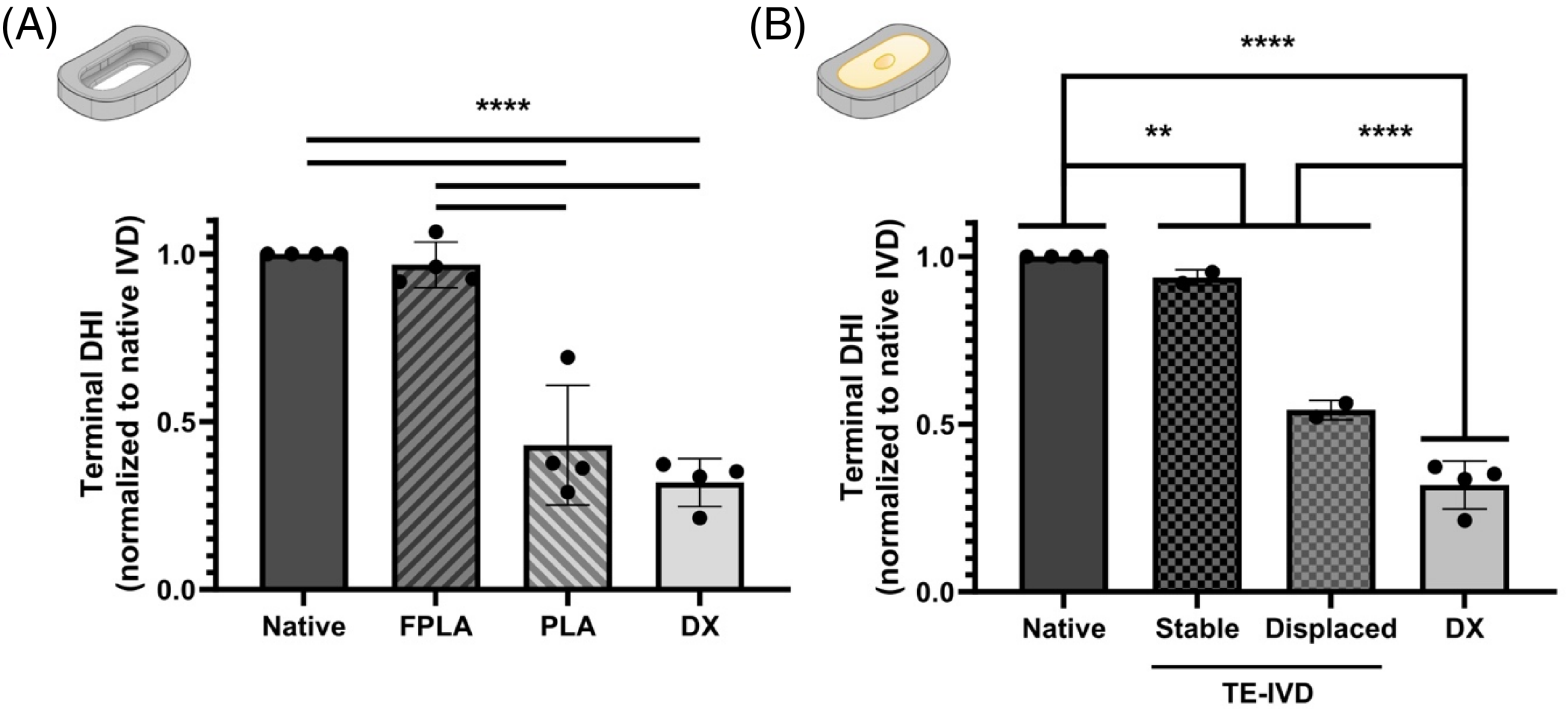
Terminal DHI measurements of empty cages or TE‐IVDs cultured in FPLA. (A) FPLA cages restored disc height to native levels and significantly improved disc height relative to PLA cages and DX levels (*p* < 0.0001). (B) Stably implanted TE‐IVDs yielded disc heights that were comparable to native IVD. Displaced TE‐IVDs yielded DHIs that were nominally lower than stably implanted constructs, but nominally greater than DX levels. TE‐IVD implantation regardless of displacement yielded disc heights that were significantly greater than DX levels (*p* < 0.0001), but significantly lower than native disc (*p* = 0.0031).

FPLA cages and stably implanted TE‐IVDs restored native disc height while PLA cages yielded DHIs which were comparable to DX levels (Figure 3). To test which material is better suited for maintaining disc height and facilitating the integration of implanted tissues, terminal DHI measurements were taken at study endpoint, after implant displacement, or after cage fracture. DHI measurements of pigs that received empty cages revealed that FPLA cages remained in place until study endpoint and restored disc height to native levels (SI. 1A). In contrast, PLA cages fractured within 4 weeks, and yielded terminal DHIs that were significantly less than native disc and not statistically different from DX levels (*p* < 0.0001) (Figure 3A). Terminal DHI measurements of pigs that received TE‐IVDs cultured in FPLA revealed that 2 implants remained stabilized in the disc space and 2 were displaced within 2 weeks following implantation (SI. 1B). Stabilized TE‐IVDs yielded disc heights which were comparable to native IVD. Displaced constructs yielded DHIs that were nominally lower than stabilized constructs and native disc, but greater than DX levels. (Figure 3B). Displaced implants were extruded anteriorly with no signs of neurological deficit or clinical complications.

### 3.3 Stably implanted TE‐IVDs supported the formation of hydrated tissues in vivo

**FIGURE 4:**
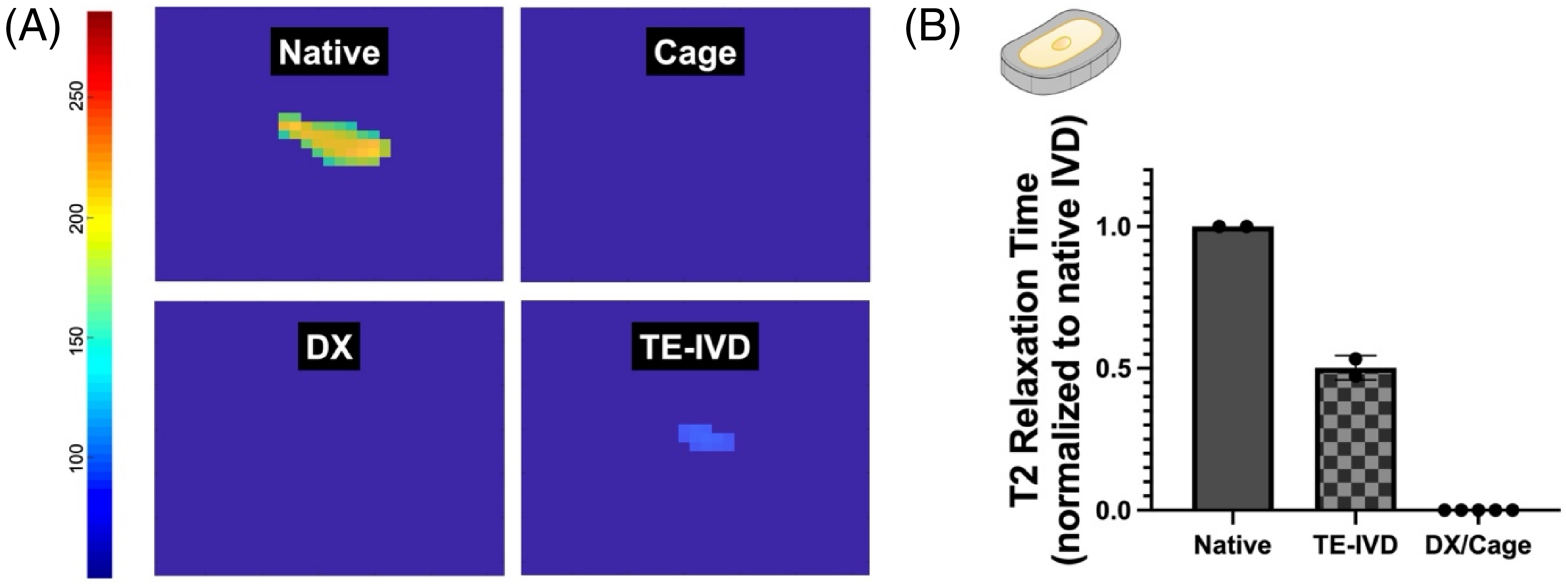
Quantitative analysis of TE‐IVD hydration via T2 MRI. (A) Representative T2 heatmaps of native disc, FPLA cages, DX controls, and levels treated with TE‐IVDs. (B) Stably implanted TE‐IVDs yielded T2 relaxation times that were nominally greater than cage implants or DX controls and nearly half the T2 relaxation time of native disc tissue.

Stably implanted TE‐IVDs supported the formation of hydrated tissues in the minipig spine, yielding tissues with half the T2 relaxation time of native disc and increasing tissue hydration relative to empty cages or DX controls (Figure 4). T2 heatmaps of stably implanted TE‐IVDs resulted in a light blue footprint while segments treated with an empty cage or left as a DX control yielded no footprint after thresholding. T2 analysis was performed using a previously established MATLAB program.^30^ Heatmaps of native disc resulted in an ellipsoidal footprint that is yellow at the center and green at the edges. Heatmaps of stably implanted TE‐IVDs resulted in a blue ellipsoidal footprint that was smaller than native disc and of uniform T2 intensity. Thresholding of cage‐treated or DX levels resulted in complete removal of T2 intensity and generated heatmaps where no signal was present (Figure 4A). The stabilization of hydrated tissues is essential for engineering a biological replacement capable of transmitting loads between vertebrae. As such, the water content of implanted TE‐IVDs was quantified via T2 MRI at study endpoint. Analysis of stabilized constructs revealed that TE‐IVDs yielded T2 relaxation times that were nearly half the relaxation time of native disc and greater than cage‐treated or DX levels (Figure 4B).

## 4 DISCUSSION

In the present study, we demonstrated that flexible materials are better suited for mechanically augmenting TE‐IVDs in the minipig spine. In vitro, FPLA cages resisted microscale damage from indentation and delayed the onset of plastic deformation when compared to PLA. In vivo, FPLA cages and stably implanted TE‐IVDs restored and maintained native disc height for 6 weeks in the minipig spine. In contrast, PLA cages fractured after 4 weeks and yielded disc heights that were similar to DX levels. TE‐IVD displacement led to a reduction in disc height but produced DHIs that were greater than DX levels. Stably implanted TE‐IVDs supported the generation of hydrated tissues in the minipig spine, and produced constructs with half the T2 relaxation time of native disc tissue.

Mechanically supporting TE‐IVDs upon implantation is crucial for their long‐term viability. As such, several studies have utilized support materials to preserve disc height and protect implants. Previous work in an ex vivo canine model externally fixed PLGA plates to adjacent vertebrae.^19^ Similarly, titanium plates were externally fixed to preserve disc height in an in vivo goat model.^14^ Although both studies restored native disc height, they utilized external fixation. External fixation limits segmental motion of the spine and increases mechanical loading of adjacent discs. Consequently, external fixation increases the risk of surgical complications including pseudoarthrosis, screw fracture, and infection.^31^, ^32^, ^33^, ^34^ To address these challenges, our group recently developed an FE model of the minipig spine to simulate in vivo failure modes of TE‐IVD support cages without external fixation.^12^ This model predicted areas of high stress on PLA support cages that align well with bony protrusions found on adjacent vertebrae. These stress concentrations led to complete collapse of the disc space and brittle fracture of PLA cages. Interestingly, the yield strength of PLA (50–70 MPa) greatly exceeds porcine intradiscal pressure (1–2 MPa).^35^, ^36^, ^37^ However, brittle materials are unable to conform to the unique anatomy of minipig vertebrae. A recent study characterized FPLA scaffolds, and found that FPLA had tunable viscoelastic properties, supported the viability of NP cells, and promoted matrix deposition.^23^ Although these results were promising, no present study has evaluated the success of FPLA support structures in vivo. Therefore, we were interested in comparing both stiff and flexible support cage materials to determine which is better suited for mitigating plastic deformation and failure.

Surprisingly, FPLA cages outperformed PLA by resisting brittle fracture from indentation and restoring native disc height for 6 weeks in vivo. In contrast to FPLA, indented PLA cages were left with a wide band of plastic deformation and evidence of brittle fracture at the indentation site (Figure 2C). Mechanical testing of printed specimens revealed that PLA cages experienced yield behavior at lower deformations than FPLA cages (Figure 2B). Our findings agree with those of another study that compared the mechanical performance of PLA and FPLA tensile specimens for anterior cruciate ligament reconstruction.^38^ This study demonstrated that PLA and FPLA specimens experienced yield behavior at ~5% and ~50% tensile strain, respectively. At 5% strain, PLA experienced 40–50 MPa of stress while FPLA experienced 1–1.5 MPa of stress. As such, the elasticity of FPLA cages allows for greater deformations in vivo without experiencing the brittle fracture seen in PLA. This suggests, that in this model there are deformations imposed on these implants that cause failure in brittle PLA, but are still in the elastic region of FPLA. Collectively, these data suggest that these failure properties, in particular, elasticity and strain to failure, are critical parameters for designing support materials for IVD implants. Uniaxial compression of cages immersed in physiologically relevant conditions revealed that both cage materials experienced an increase in stress at 80% strain after immersion (Figure 2D). Immersion of FPLA cages led to smaller changes in the stress measured at 80% strain when compared to PLA cages. Interestingly, our findings agree with those of a related study that measured the dynamic moduli of degraded PLA and FPLA scaffolds.^23^ This study demonstrated that 3D‐printed FPLA possessed superior stability against hydrolysis when compared to 3D‐printed PLA. Moreover, their degradation studies revealed that FPLA scaffolds experienced minimal changes in scaffold mass or mechanics for up to 34 weeks while PLA scaffolds experienced brittle failure at 26 weeks.

Collectively, these findings demonstrate that FPLA is better suited for preventing the onset of yield behavior. These results motivated us to investigate whether flexible or stiff TE‐IVD support materials could restore native disc height in vivo. In the minipig spine, FPLA cages and stably implanted TE‐IVDs outperformed PLA by yielding greater disc heights (Figure 3). Disc height measurements of TE‐IVDs revealed that displaced TE‐IVDs yielded disc heights that were nominally greater than PLA and DX levels. Interestingly, another study evaluated the mechanical endurance of a flexible, mono‐unit cervical disc implant.^39^ After 6 million cycles of loading, disc height at the level of prosthesis (8.4 mm) was comparable to disc height before loading (8.9 mm). These findings support the feasibility of using flexible support materials to maintain disc height for extended periods of time or after rigorous use. Altogether, these results demonstrate that flexible implants are superior for restoring native disc height and demonstrate a therapeutic benefit from implanting a TE‐IVD support structure.

We were also interested in investigating whether FPLA cages would support the formation of hydrated tissues *in vivo*. The generation of hydrated tissues is essential for facilitating load transmission between vertebrae. Therefore, tissue hydration was quantified from T2 MRI scans of stabilized TE‐IVDs. Stably implanted TE‐IVDs produced constructs with half the T2 relaxation time of native disc tissue, indicating that these constructs were half as hydrated (Figure 4). This amount of hydration is consistent with previous findings in both canine^13^ and caprine^14^ animal models. T2 relaxation times of human IVD at end‐stage degeneration vary between 35 and 60 ms, with complete collapse of the disc space resulting in full loss of T2 signal. Meanwhile, relaxation times of healthy discs, with a Pfirrmann grade of 1, vary between 100 and 175 ms.^40^, ^41^, ^42^, ^43^ At half the T2 relaxation time of native disc, stably implanted TE‐IVDs possess superior hydration when compared to fully degenerated or collapsed discs. Collectively, this demonstrates that flexible cages support the formation of hydrated tissues in the minipig spine.

Although this study was the first to compare stiff and flexible TE‐IVD support structures in vivo, inherent limitations are the low sample size and study duration. Increasing the sample size may demonstrate that the nominal difference in disc height between PLA cages and DX levels is statistically significant. Moreover, increasing the sample size would allow for direct statistical comparisons between stably implanted and displaced TE‐IVDs as well as a statistical analysis of T2 intensity. Increasing the duration of this study would allow us to assess tissue integration after degradation of support materials. Another limitation of this study was that implant displacement occurred in half of the implants. Previous work addressed this limitation by utilizing a plating system to prevent displacement.^14^, ^19^ However, these studies prioritized choosing a support material with high stiffness rather than elasticity, thus introducing the risk of brittle fracture or requiring a revision surgery to remove the support material once integration is achieved. Future studies may address these issues by implementing a flexible and resorbable plating system to enhance implant retention. This study would also be strengthened by including histology. Motion segments of cage, DX, and TE‐IVD levels were prepared, but the presence of a support cage at the bone‐disc interface led to sectioning difficulties. Although histology would provide greater insight on the structure and composition of TE‐IVDs, outcomes from this study still point to the benefits of mechanically augmenting engineered tissues with flexible support materials.

This study is the first to use flexible materials to mechanically support TE‐IVDs in a large animal model. Although stiffer materials have been a mainstay for providing mechanical support in orthopedic applications, results from this study demonstrate the importance of flexibility when mechanically augmenting TE‐IVDs. Flexibility is essential for preventing brittle fracture, restoring disc height, and supporting the formation of hydrated tissues in vivo. Together, these findings highlight the importance of choosing a material with greater elasticity to ensure the long‐term success of TE‐IVDs.

## AUTHOR CONTRIBUTIONS

**Alikhan Fidai**: Conceptualization, data curation, performance of in vitro studies and in vivo analysis, project administration, writing–review and editing. **Byumsu Kim**: Conceptualization, data curation, writing–review and editing. **Marianne Lintz**: Conceptualization, data curation, writing–review and editing. **Sertac Kirnaz**: Data curation, project administration, surgical expertise, writing–review and editing. **Pravesh Gadjradj**: Project administration, surgical expertise, writing–review and editing. **Blake I. Boadi**: Project administration, writing–review and editing. **Maho Koga**: Writing–review and editing. **Ibrahim Hussain**: Conceptualization, surgical expertise, supervision, writing–review and editing. **Roger Härtl**: Conceptualization, surgical expertise, supervision, writing–review and editing. **Lawrence J. Bonassar**: Conceptualization, supervision, writing–review and editing.

## FUNDING INFORMATION

This work was partially funded by the Daedalus Fund and the Weill Cornell Medicine Clinical and Translational Science Center TL1 Training Grant.

## CONFLICT OF INTEREST STATEMENT

Lawrence J. Bonassar, Ibrahim Hussain, and Roger Härtl are consultants for 3DBio Therapeutics Corp., Lawrence J. Bonassar is co‐founder of 3DBio Therapeutics Corp. Remaining authors declare no conflict of interests.

## Supporting information


**Data S1.** Supporting Information.
