# Supplementary material for: Flexible support material maintains disc height and supports the formation of hydrated tissue engineered intervertebral discs in vivo
Source: JOR Spine. 2024 Aug 5;7(3):e1363. doi: 10.1002/jsp2.1363 (PMC11299905; doi:10.1002/jsp2.1363)
Supplement: Supplementary file 1 — Data S1. Supporting Information. [file JSP2-7-e1363-s001.docx]

**SUPPORTING INFORMATION**

**
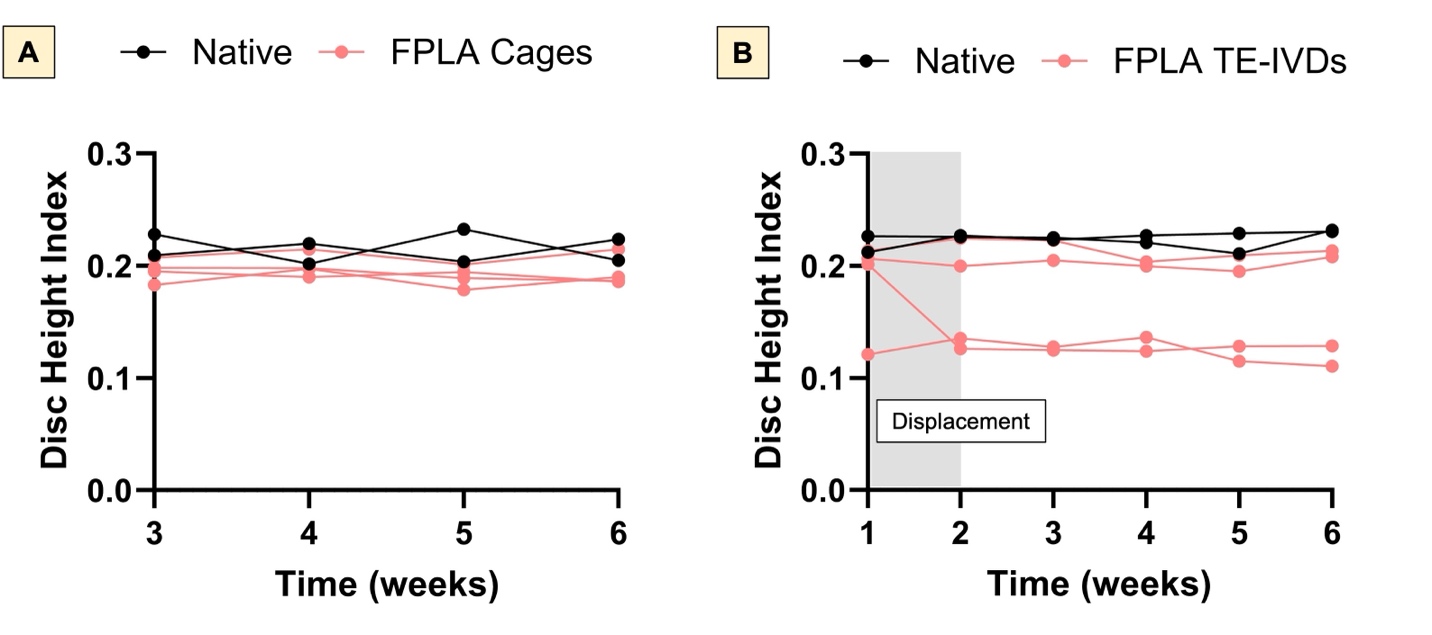
**

**Supporting Information 1.** DHI measurements of FPLA implants over time. **A**) FPLA cages were stably implanted until study endpoint. **B**) TE-IVDs cultured in FPLA were either displaced within 2 weeks of implantation or stably implanted until study endpoint.
